# Berberine alters gut microbial function through modulation of bile acids

**DOI:** 10.1186/s12866-020-02020-1

**Published:** 2021-01-11

**Authors:** Patricia G. Wolf, Saravanan Devendran, Heidi L. Doden, Lindsey K. Ly, Tyler Moore, Hajime Takei, Hiroshi Nittono, Tsuyoshi Murai, Takao Kurosawa, George E. Chlipala, Stefan J. Green, Genta Kakiyama, Purna Kashyap, Vance J. McCracken, H. Rex Gaskins, Patrick M. Gillevet, Jason M. Ridlon

**Affiliations:** 1 Institute for Health Research and Policy, University of Illinois Chicago, Chicago, IL USA; 2 Cancer Education and Career Development Program, University of Illinois, Chicago, IL USA; 3 Department of Animal Sciences, University of Illinois Urbana-Champaign, Urbana, IL USA; 4 Division of Nutritional Sciences, University of Illinois Urbana-Champaign, Urbana, IL USA; 5 Carl R. Woese Institute for Genomic Biology, University of Illinois Urbana-Champaign, Urbana, IL USA; 6 Structural and Computational Biology Research Unit, European Molecular Biology Laboratory, Heidelburg, Germany; 7 Center for Microbiome Analysis, George Mason University, Manassas, VA USA; 8 Junshin Clinic Bile Acid Institute, Meguro-Ku, Tokyo, 152-0011 Japan; 9 School of Pharmaceutical Sciences, Health Sciences University of Hokkaido, Tobetsu, Japan; 10 University of Illinois Chicago Research Resources Center, University of Illinois Chicago, Chicago, IL USA; 11 Department of Internal Medicine, School of Medicine, Virginia Commonwealth University, Richmond, VA USA; 12 Department of Internal Medicine, Mayo Clinic, Rochester, MN USA; 13 Department of Biological Sciences, Southern Illinois University Edwardsville, Edwardsville, IL USA; 14 Department of Pathobiology, University of Illinois Urbana-Champaign, Urbana, IL USA; 15 Cancer Center of Illinois, University of Illinois Urbana-Champaign, Urbana, IL USA; 16 Department of Microbiology and Immunology, School of Medicine, Virginia Commonwealth University, Richmond, VA USA

**Keywords:** Berberine, Bile acids, Gnotobiotic mice, Gut bacteria, Network analysis, Nutraceutical, RNA-Seq

## Abstract

**Background:**

Berberine (BBR) is a plant-based nutraceutical that has been used for millennia to treat diarrheal infections and in contemporary medicine to improve patient lipid profiles. Reduction in lipids, particularly cholesterol, is achieved partly through up-regulation of bile acid synthesis and excretion into the gastrointestinal tract (GI). The efficacy of BBR is also thought to be dependent on structural and functional alterations of the gut microbiome. However, knowledge of the effects of BBR on gut microbiome communities is currently lacking. Distinguishing indirect effects of BBR on bacteria through altered bile acid profiles is particularly important in understanding how dietary nutraceuticals alter the microbiome.

**Results:**

Germfree mice were colonized with a defined minimal gut bacterial consortium capable of functional bile acid metabolism (*Bacteroides vulgatus, Bacteroides uniformis, Parabacteroides distasonis, Bilophila wadsworthia, Clostridium hylemonae, Clostridium hiranonis, Blautia producta*; B4PC2). Multi-omics (bile acid metabolomics, 16S rDNA sequencing, cecal metatranscriptomics) were performed in order to provide a simple in vivo model from which to identify network-based correlations between bile acids and bacterial transcripts in the presence and absence of dietary BBR. Significant alterations in network topology and connectivity in function were observed, despite similarity in gut microbial alpha diversity (*P =* 0.30) and beta-diversity (*P* = 0.123) between control and BBR treatment. BBR increased cecal bile acid concentrations, (*P <* 0.05), most notably deoxycholic acid (DCA) *(P* < 0.001). Overall, analysis of transcriptomes and correlation networks indicates both bacterial species-specific responses to BBR, as well as functional commonalities among species, such as up-regulation of Na^+^/H^+^ antiporter, cell wall synthesis/repair, carbohydrate metabolism and amino acid metabolism. Bile acid concentrations in the GI tract increased significantly during BBR treatment and developed extensive correlation networks with expressed genes in the B4PC2 community.

**Conclusions:**

This work has important implications for interpreting the effects of BBR on structure and function of the complex gut microbiome, which may lead to targeted pharmaceutical interventions aimed to achieve the positive physiological effects previously observed with BBR supplementation.

**Supplementary Information:**

The online version contains supplementary material available at 10.1186/s12866-020-02020-1.

## Background

There is considerable interest in the utilization of dietary components to modulate the gut microbiome in a manner that improves human and animal health. This is especially true of plant-based nutraceutical compounds that have been used for millennia in traditional human societies. Nutraceuticals are now being studied to determine their efficacy in microbiome-based health outcomes and their mechanism of action under controlled conditions. Berberine (BBR) is an isoquinoline alkaloid nutraceutical compound found in certain roots (*Rhizoma coptidis*) and berries (*Berberis vulgaris, Coptis chinensis*) that is traditionally utilized to treat diarrhea through its anti-microbial action [1]. Berberine also exerts lipid-lowering effects through activation of the AMP-activated protein kinase signaling pathway and increased expression of low-density lipoprotein receptor in the liver [2, 3]. Additionally, BBR functions to reduce serum cholesterol by up-regulating the conversion of cholesterol into bile acids which are excreted at higher levels in feces [4, 5]. The biotransformation of BBR by gut bacteria appears to be crucial for absorption across the gut epithelium [6, 7]. Because BBR has low bioavailability outside the GI tract, the beneficial properties of BBR are thought to be due to local GI effects on the gut microbiota [6, 8, 9]. Recent reports detail alterations in gut microbiome function caused by oral BBR administration in hamsters [4], rats [8], and mice [9] including decreased taxonomic richness and enrichment of bacteria that produce short chain fatty acids (SCFA). However, detailed transcriptional responses of gut bacteria to BBR treatment in vivo have yet to be reported. Moreover, since bile acids are themselves antimicrobial [10], and because bile acid concentrations are increased in response to BBR treatment, determining bile acid-dependent correlations with microbial gene expression is also important. Investigations into these responses are predicted to provide testable hypotheses that will enable future examinations of how BBR alters bacterial structure and function, and how bacteria adapt in the short-term to antimicrobial dietary compounds such as BBR, particularly in response to increased influx of intestinal bile acids.

A simple in vivo gut community model is particularly effective in measuring the effects of single dietary nutraceuticals on genome-wide microbial gene expression, particularly with microbes that are typically found in low abundance. For this we developed a microbial community modified from Narushima et al. composed of bacteria commonly found in the human GI tract that are capable of bile acid metabolism [11]. We have recently reported in vitro bile acid-induced transcriptional changes in low abundant bile acid metabolizing bacteria including *C. scindens* [12], *C. hylemonae*, and *C. hiranonis* [13]. Moreover, we determined the in vivo transcriptional profile of *C. hylemonae* and *C. hiranonis* in the mouse cecum in the presence of *Ba. uniformis, Ba. vulgatus, Bi. wadsworthia, P. distasonis*, and *Bl. producta* [13]. We have shown that this small consortium, termed ‘B4PC2’, is capable of completely converting host taurine-conjugated bile acids to unconjugated bile acids and secondary bile acids such as ursodeoxycholic acid (UDCA), DCA, and lithocholic acid (LCA). Here, we examine individual bacterial responses to BBR, and show that network correlations among host liver, cecal, and serum bile acids and bacterial transcript abundances change significantly with oral administration of BBR.

## Results

### Effect of berberine on global bile acid metabolome

**Fig. 1:**
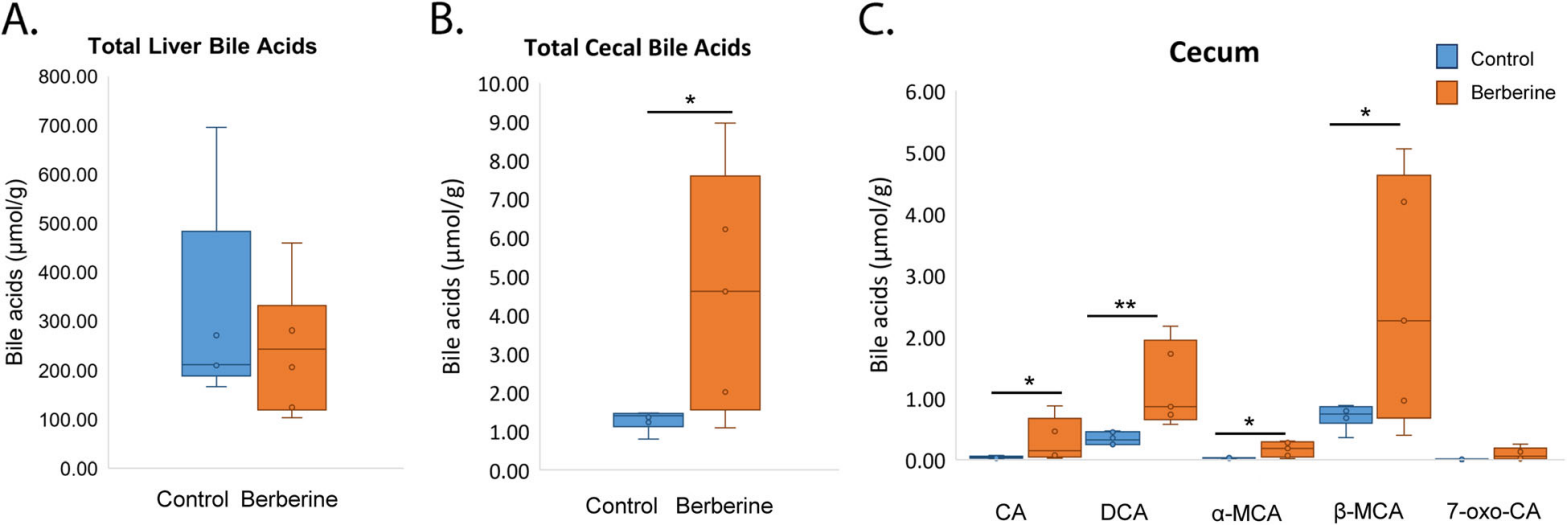
Berberine increases cecal total bile acids and deoxycholic acid. **a**. Box-plot of total bile acids in the liver between control and berberine-treated mice. **b**. Total bile acids in cecum between control and berberine-treated mice. **c**. Selected bile acids in cecum between control and berberine-treated mice. *P* < 0.05 (*); *P* < 0.01 (**)

Since previous reports have indicated that BBR affects hepatic lipids and cholesterol by increasing bile acid excretion into the large intestine [4, 6, 14], the global bile acid metabolome was examined in control and BBR treated gnotobiotic mice. Total liver bile acid concentrations were not significantly different between control and BBR treated mice (*P =* 0.5283) (Fig. 1a). Significant compositional differences between bile acids in BBR treated and control liver and serum were not observed; however, microbial secondary bile acid products such as DCA, taurodeoxycholic acid (TDCA), and taurolithocholic acid (TLCA) were observed, indicating functional bile acid metabolism by the B4PC2 consortium in the GI tract (Fig. S1, S2 and S3). By contrast, a significant increase in total cecal bile acids (4.57 ± 1.42 μmol/g vs. 1.29 ± 0.106 μmol/g; *P* < 0.05) (Fig. 1b), and cecal bile acid composition was observed after BBR treatment relative to control (Fig. 1c & S4). We determined that total bile acids (*P* = 0.17;0.85) and DCA (*P* = 0.098; 0.23) in the liver and cecum did not differ between males and females, respectively. These disparate responses to BBR treatment observed in liver, serum, and cecum suggest that BBR increases fecal loss of bile acids with concomitant increased synthesis of bile acids in order to maintain baseline liver bile acid concentrations.

Functional bile acid metabolism in the cecum by the human gut B4PC2 community was evident in both control and BBR treatment groups. Deconjugation of taurine-conjugated bile acids was nearly complete. DCA and LCA, the end-products of the bile acid 7α-dehydroxylation pathway encoded by *C. hylemonae* and *C. hiranonis* [15], were major metabolites in the cecum (Fig. S4). Conversion of CDCA to α- and β-muricholic acid (MCA) was observed (Fig. S4), however little conversion of MCA to murideoxycholic acid (MDCA) and ω-MCA was detected. This confirmed the mice in this study housed a microbial consortium of human bacteria, as only limited MDCA has been shown to be generated by the host [16], and human mixed fecal bacteria and bile acid 7α-dehydroxylating clostridia appear to be unable to metabolize MCA to MDCA and ω-MCA [12, 15, 17].

### Cecal composition of the B4PC2 human microbial consortium in control and berberine treated gnotobiotic mice

**Fig. 2:**
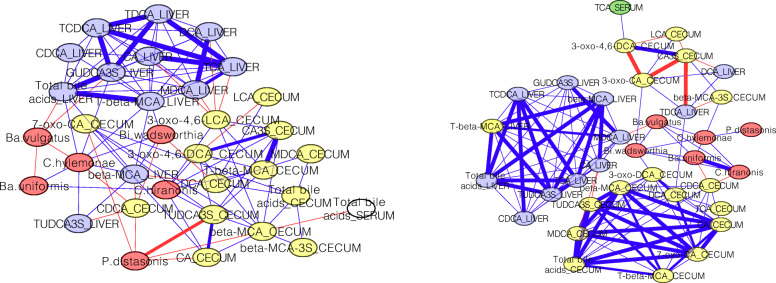
Network interactions between bacterial taxa in the cecum and bile acids in the liver, serum, and cecum on control diet and berberine treatment. Nodes are as follows: Liver bile acids (blue ovals), cecal bile acids (yellow ovals), cecal bacteria (red ovals). Thick lines represent correlations > 0.9, thin lines represent correlations < 0.9

To determine whether the B4PC2 consortium was established in both control and BBR treated mice a microbial analysis of cecal content was performed. Sequencing of 16S rDNA resulted in 53,456 ± 3743 reads for control ceca and 53,529 ± 3441 reads for BBR treated ceca. Overall diversity of both control and BBR mice were consistent with the inoculated consortium indicating successful maintenance of the germ-free environment (Fig. S5). To examine whether the composition of this bile-tolerant community is altered by BBR treatment, diversity analyses were performed on 23,900 rarefied reads from the 16S rDNA dataset. Non-metric multidimensional scaling (NMDS) and Analysis of Similarities (ANOSIM) tests of differences in beta-diversity resulted in *R* = 0.141 and *P* = 0.123 (999 permutations) indicating diversity between samples was not significant (Fig. S5). Shannon index (alpha diversity) was not significantly different between groups (*P =* 0.30; Mann-Whitney test) (Fig. S6). Microbiome abundances were not significantly different between sexes [*Bacteroides* (*P* = 0.60), *Parabacteroides* (*P* = 0.19), *Clostridiaceae* (*P* = 0.63), *Bilophila* (*P* = 0.79)], so we did not separate out sex in further analyses. We next performed network correlation analyses on bile acids in the cecum, serum, and liver with abundances of B4PC2 consortium members. Substantial network topological changes between bile acids, and microbial taxa were observed between control and BBR networks (Fig. 2**;** Supplementary Dataset). These analyses indicate that the B4PC2 consortium was similarly established in control and BBR treated gnotobiotic mice, suggesting that BBR mechanisms of action are not related to composition changes in this bile acid metabolizing microbial community.

### Direct effects of berberine and bile acid concentrations on gene expression by the B4PC2 consortium

Given observed topological changes between the bile acid metabolome and the B4PC2 consortium networks between treatments (Fig. 2), RNAseq was performed on cecal content collected from control and BBR treated mice. Network correlation analyses between transcriptomic and metabolomic data were created for each member of the B4PC2 consortium in order to decern whether differences in gene expression are in response to bile acid concentration or direct effect of BBR treatment. Results for each B4PC2 member are highlighted in the following sections.

### Bilophila wadsworthia

**Fig. 3:**
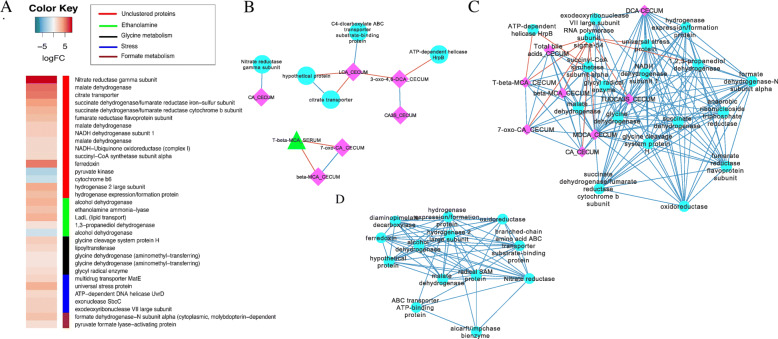
Effect of berberine on *Bilophila wadsworthia* on in vivo gene expression and network interactions. **a**. Heat map of differential gene expression (log_2_FC > (−)0.58; *P* < 0.05) for *B. wadsworthia* grouped by function. **b**. Network interactions between *B. wadsworthia* cecal gene expression and bile acid profile from cecum, liver, and serum in control group. **c**. Network interactions between *B. wadsworthia* cecal gene expression and bile acid profile from cecum, liver, and serum in berberine-treated group. **d**. Sub-network showing interactions between DCA in the cecum and gene expression as well as gene expression interaction with nitrate reductase. Data points with Spearman correlations < 0.7 and a *P* values < 0.05 are displayed

Berberine treatment resulted in significant differential expression of 123 genes (74 up-regulated; 49 down-regulated) (Fig. 3a**;** Supplementary Dataset). Transcriptome data indicate that in presence of BBR, *B. wadsworthia* imports bacterial membrane lipids (phosphatidylethanolamine) (*LadL*; 3.29 log_2_FC, *P* = 0.01), degrades ethanolamine to acetaldehyde and ammonia (ethanolamine ammonia-lyase), and converts acetaldehyde to ethanol (*adh*; 3.29 log_2_FC, *P* = 2.46E-3). High relative expression of group 1b NiFeSe hydrogenase was observed (3.56 log_2_FC, *P* = 3.66E-4), which is involved in liberation of electrons for formate, sulfite, and nitrate respiration. Pyruvate-formate lyase and formate dehydrogenase were highly expressed in *Bilophila* during BBR treatment. However, the gene most highly-expressed was nitrate reductase γ-subunit (8.04 log_2_FC, *P* = 7.07E-06). The other two most highly expressed genes were citric acid cycle enzyme malate dehydrogenase (5.43 log_2_FC, *P* = 1.04E-5) and citrate transporter (5.11 log_2_FC, *P* = 1.04E-4). Additional citric acid cycle genes and respiratory complex genes are significantly up-regulated by BBR (Fig. 3a**;** Supplementary Dataset). In addition, a gene involved in efflux of toxic substances (*matE*) was significantly up-regulated by BBR (1.72 log2FC; *P* = 0.01), which may indicate export of BBR by this gene product.

Berberine treatment significantly affected the topology and complexity of networks between *Bilophila* gene expression and bile acid profiles in liver, serum, and cecum (Fig. 3b & c). Many of the gene expression networks, sparse in the control and unconnected with bile acids, become highly interconnected during BBR treatment and relate either directly or indirectly to increased bile acid concentrations. Notably, universal stress protein (*uspA*) was highly expressed in the BBR group relative to control (3.36 log_2_FC; *P* = 0.05) as were genes involved in DNA recombination and repair (*sbcC*, *xseA*, *uvrD*). The expression of *uspA* revealed a strong positive correlation with cecal bile acids, including DCA (r = 1.0; *P* = 0.0), tauroursodeoxycholic acid-3-sulfate (TUDCA3S) (r = 0.97; *P* = 0.0), and MDCA (r = 0.97; *P* = 0.0). DCA also correlated strongly with a recently described glycyl radical enzyme (T370_R50117375; r = 1.0; *P* = 0.0) involved in sulfide formation from taurine (Fig. 3d) [18]. DCA and the glycyl radical enzyme shared strong positive correlation with NADH dehydrogenase subunit 1 (T370_R50109290; r = 1.0; *P* = 0.0) as well as direct positive correlations with glycine dehydrogenase (T370_R50113235; r = 1.0; *P* = 0.0). In control mice, nitrate reductase γ-subunit is positively correlated with cecal CA (r = 0.93; *P* = 0.001) (Fig. 3b**;** Supplementary Dataset); whereas there are no direct correlations between cecal bile acids and γ-subunit in BBR treatment (Fig. 3c**;** Supplementary Dataset).

### Bacteroides uniformis

**Fig. 4:**
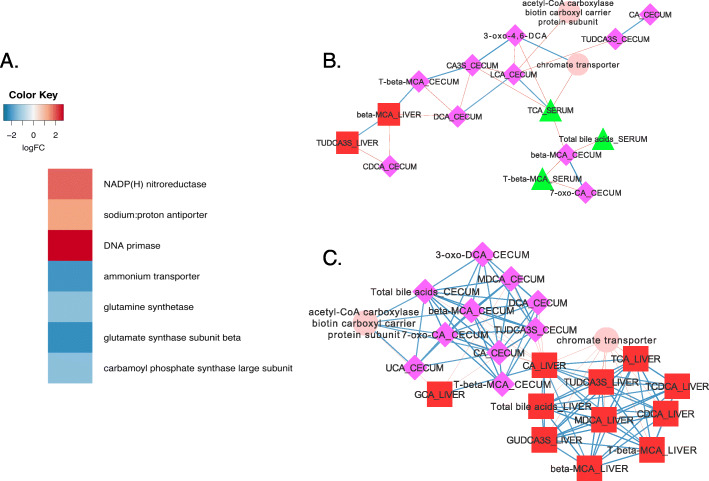
Response of *Bacteroides uniformis* to berberine treatment. **a**. Heat map of differentially expressed genes (log_2_FC > (−)0.58; *P* < 0.05) by *B. uniformis*. **b**. Network interactions in control mice. **c**. Network interactions in berberine-treated mice. Nodes are as follows: bacterial genes expressed (pink circles), cecal bile acids (fuschia squares) and serum bile acids (green triangles). Data points with Spearman’s correlations < 0.7 and a *P* values < 0.05 are displayed

Seventeen genes were significantly differentially regulated in *B. uniformis* by BBR (Fig. 4a**;** Supplementary Dataset). Two genes were identified whose expression correlated to bile acids: the highly up-regulated NAD(P)H nitroreductase (ERS852554_00867; 2.49 log_2_FC; *P* = 0.02), as well as the Na^+^/H^+^ antiporter (1.34 log_2_FC; *P* = 0.02). A high degree of positive connectivity was observed between total cecal bile acids (r = 0.8; *P* = 0.046), and primary unconjugated bile acids including β-MCA (r = 0.8; *P* = 0.046), UCA (r = 0.87; *P* = 0.015) and 7-oxo-CA (r = 0.8; *P* = 0.046), as well as expression of acetyl-CoA carboxylase biotin carboxyl carrier protein which was induced by BBR treatment (BLV12_RS03955; 2.47 log_2_FC *P* = 4.51E-03; FDR = 0.48) (Fig. 4b & c**;** Supplementary Dataset). Chromate transporter (BLV12_RS04500; Log_2_FC = 3.06; *P* = 0.01; FDR = 0.58) was negatively correlated with total bile acids in the liver (r = − 0.9; *P* = 0.006) and individual conjugated, sulfated, and primary bile acids (r = − 0.9 to − 1.0; *P* = 0.006 to *P* < 0.001).

### Bacteroides vulgatus

**Fig. 5:**
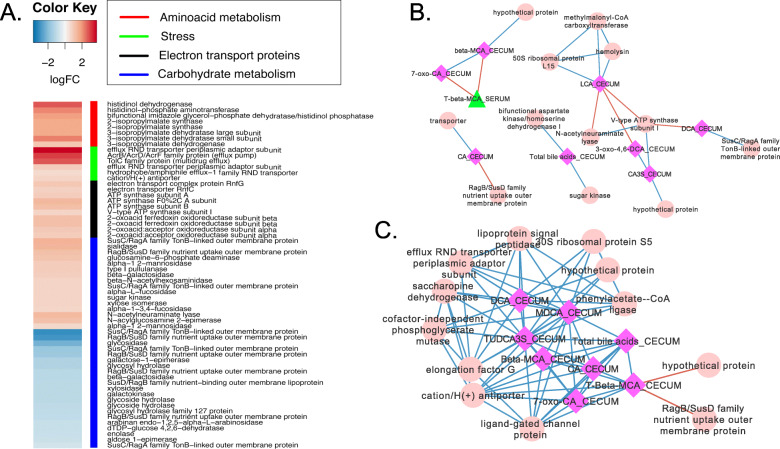
Network analysis of *Bacteroides vulgatus* response to berberine. **a**. Heat map of differentially expressed genes (log_2_FC > (−)0.58; *P* < 0.05) by *B. vulgatus*. **b**. Network interactions in control mice. **c**. Network interactions in berberine-treated mice. **d**. Sub-network of interactions with DCA in the cecum. Nodes are as follows: bacterial gene expression (pink circles), liver bile acids (red squares), serum bile acids (green triangles), cecal bile acids (fuschia squares). Data points with Spearman’s correlations < 0.7 and a *P* values < 0.05 are displayed

BBR differentially altered expression of 105 genes in *B. vulgatus* (Fig. 5a**;** Supplementary Dataset). Most notably, BBR increased the expression of a polycistronic operon encoding predicted multidrug efflux pump subunits—periplasmic adaptor subunit efflux resistance-nodulation-division (RND) (3.72 log_2_FC; *P* = 7.23E-08; FDR = 1.23E-05), AcrB/AcrD/AcrF (3.00 log2FC; *P* = 5.54E-07; FDR = 6.87E-05), and TolC (2.71 log_2_FC; *P* = 1.6E-07; FDR = 2.26E-05). Expression of the efflux RND transporter periplasmic subunit (BVU_RS20445) was positively associated with DCA in the cecum (r = 1.0; *P* < 0.001) as well as cecal MDCA (r = 0.97; *P* = 0.0) and TUDCA-3S (r = 0.97; *P* < 0.001). In addition, a gene encoding a predicted cation/H(+) antiporter was positively correlated with total cecal bile acids (r = 1.0; *P* < 0.001) (Fig. 5b, c and d; Supplementary Dataset).

BBR induced increased expression of sialidase (1.44 log_2_FC, *P* = 2.93E-05, FDR = 1.81E-03) and other genes involved in mucin degradation including *N*-acetylneuraminate lyase (1.33 log_2_FC, *P* = 9.64E-04, FDR = 0.04), *N*-acylglucosamine 2-epimerase (1.18 log_2_FC, *P =* 3.95E-08, FDR = 0.08), α-1,2-mannosidase (0.93 log_2_FC, *P =* 1.64E-03 FDR = 0.05), α-L-fucosidase (0.75 log_2_FC, *P =* 0.02, FDR = 0.23), and α-1,2-C3/C4-fucosidase (0.70 log_2_FC; *P* = 0.02; FDR = 0.23). SusC and SusD outer membrane protein encoding genes, involved in binding and uptake of carbohydrates, were observed in the correlation network in the BBR treated group. In particular, BVU_1844 was differentially expressed in the BBR group (1.18 log_2_FC; *P* = 0.03; FDR = 0.31) and negatively correlated with cecal T-β-MCA (r = − 0.97; *P* < 0.001). These data may indicate a “ramping up” of carbohydrate metabolism and may explain the significant increase in total SCFA levels reported previously during BBR intake [8].

### Parabacteroides distasonis

**Fig. 6:**
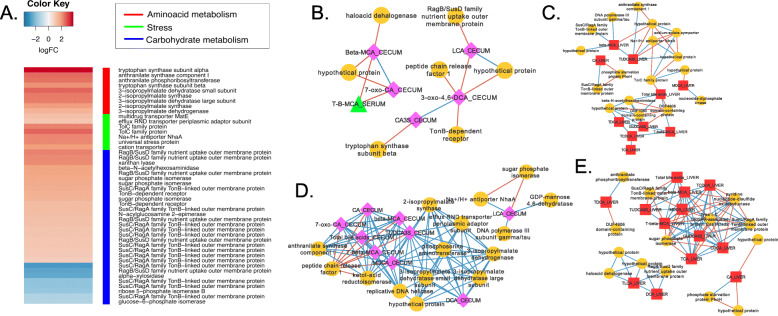
Effect of berberine on *P. distasonis* gene expression-bile acid network interactions. **a**. Heat map of differentially expressed genes (log_2_FC > (−)0.58; *P* < 0.05) by *P. distasonis* in the mouse cecum between control diet and berberine treatment. **b**. Network of cecal bile acid (pink diamonds), serum bile acids (green triangle), and cecal bacterial gene expression (orange circules) in control ceca. **c**. Correlations between liver bile acids (red squares) and cecal gene expression in control ceca. **d**. Network of cecal bile acids, serum bile acids, cecal bacterial gene expression in mouse berberine-treated ceca. **e**. Correlations between liver bile acids and cecal gene expression during berberine-treatment. Data points with Spearman’s correlations < 0.7 and a *P* values < 0.05 are displayed

Two operons encoding predicted tryptophan (BDI_RS02910-BDI_RS02940) and leucine biosynthesis (BVU_RS10160-BVU_RS10180; BVU_RS12860-BVU_RS12880) pathways were among the most highly differentially up-regulated genes in *P. distasonis* in the presence of BBR (Fig. 6a**;** Supplementary Dataset). Genes encoding a predicted efflux RND transporter periplasmic adaptor (BDI_RS01695; 2.33 log_2_FC; *P* = 1.50E-04; FDR = 0.01) and TolC (BDI_RS00690; 2.30 log_2_FC; *P* = 2.04E-06; FDR = 1.37E-03) were also highly expressed, which may reflect adaptation to increased bile salt concentrations in response to BBR. Network analysis showed sparse associations between transcripts and cecal bile acids in control mice ceca and liver (Fig. 6b & c**;** Supplementary Dataset), but tight interconnections between cecal and liver bile acids and transcripts after BBR treatment (Fig. 6d & e). While TolC expression was not correlated with cecal bile acids, efflux RND transporter had strong positive correlations with cecal DCA (r = 1.0; *P* < 0.001), TUDCA3S (r = 0.97; *P* < 0.001), and MDCA (r = 0.97; *P* < 0.001) in the BBR group (Fig. 6d). Positive correlations were also observed between cecal bile acids and genes involved in leucine and tryptophan biosynthesis (Fig. 6d). Also notable is the positive association between LCA in the cecum and the Na+/H+ antiporter NhaA (BDI_R503835; 1.53 log_2_FC; *P* = 4.38E-03; FDR = 0.12; r = 0.97; *P* < 0.001) (Fig. 6d). As in *B. vulgatus,* several SusC/SusD membrane associated protein genes were also differentially regulated by BBR and correlate directly or indirectly with bile acids in the cecum and liver (Fig. 6d & e**)**. Interestingly, *P. distasonis* is observed to differentially express multidrug transporter *matE* (WP_011966429.1; 2.60 log_2_FC; *P* = 5.51E-05; FDR = 0.04), which does not correlate with bile acids and may indicate an export protein important for removing intracellular BBR. Indeed, MATE transporters have been shown previously to catalyze xenobiotic compound efflux in a Na + or H+ dependent manner [19, 20].

### Clostridium hiranonis

**Fig. 7:**
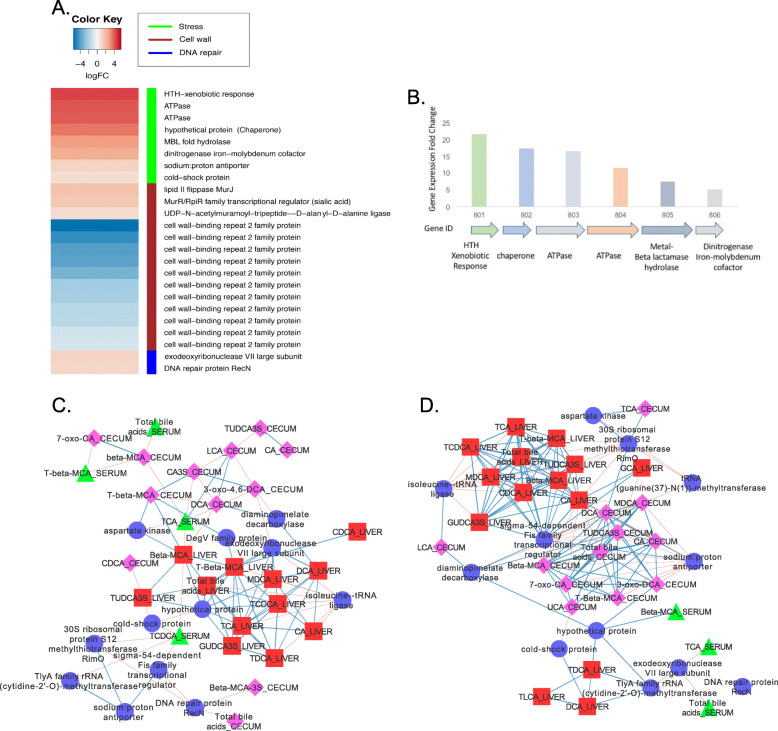
*Clostridium hiranonis* expresses a gene cluster encoding a xenobiotic response transcription factor during berberine treatment. **a**. Heat map of differentially expressed genes (log_2_FC > (−)0.58; *P* < 0.05) by *C. hiranonis* in the mouse cecum between control diet and berberine treatment. **b**. Organization and gene fold change of a gene cluster highly upregulated by berberine. **c**. Network displaying interactions between expressed genes and bile acid metabolome in control mice. **d**. Network displaying interactions between expressed genes and bile acid metabolome in berberine-treated mice. Data points with Spearman’s correlations < 0.7 and a *P* values < 0.05 are displayed

The most highly expressed genes in response to BBR in *C. hiranonis* were a 6 ORF polycistron encoding a helix-turn-helix xenobiotic response protein, chaperone, ATPase1, ATPase2, metallo-beta-lactamase fold hydrolase, and dinitrogenase iron-molybdenum cofactor (4.4 to 2.36 log2FC, *P* = 5.18E-05 to *P* = 0.024) (Fig. 7a & b**;** Supplementary Dataset). Genes involved in peptidoglycan synthesis (*murJ* and *murF*) and maintenance of the cell-wall were also significantly up-regulated by BBR. Stress-induced genes, genes involved in DNA repair (*recN* 0.98 Log2FC; *P* = 0.021), and the exodeoxyribonuclease VII large subunit (1.19 Log2FC; *P* = 0.015) were also induced by BBR treatment (Fig. 7a). The expression of exodeoxyribonuclease VII large subunit correlated positively with DCA in the liver (r = 0.95; *P* < 0.001) and less-positive correlations were observed for other liver bile acids (MDCA, T-β-MCA, TCA, TCDCA) and negatively with 3-dehydro-4,6-CA in the control cecum (r = − 0.89; *P* = 0.008) (Fig. 7c & d**;** Supplementary Dataset). Expression of *recN* negatively correlated with total cecal bile acids (r = − 0.89; *P* = 0.004) and σ^54^-dependent Fis family transcriptional regulator (r = − 0.9; *P* = 0.006). Interestingly, treatment with BBR changed this interaction (Fig. 7d; Supplementary Dataset). The expression of exodeoxyribonuclease VII large subunit was not contingent on σ^54^-dependent Fis family transcriptional regulator, but was negatively correlated with serum TCA (r = − 0.86; *P* = 0.017) and total serum bile acids (r = − 0.86; *P* = 0.017). Expression of σ^54^-dependent Fis family transcriptional regulator correlated positively with liver bile acids (r = 0.8 to 1.0; *P* values from < 0.05 to < 0.001), and Na^+^/H^+^ antiporter which was itself positively correlated with CA in the liver (r = 0.8; *P <* 0.05) but negatively correlated with numerous cecal bile acids (r = − 0.89 to 0.0; *P* values from 0.007 to < 0.001). It is possible that by importing protonated bile acids, it is not necessary to exchange ions, and expression of the Na^+^/H^+^ antiporter may decrease.

A putative adhesin was also significantly expressed in the presence of BBR (2.68 Log2FC; *P* = 2.57E-03). Numerous copies of genes encoding putative cell wall-binding repeat 2 family protein were significantly down-regulated by BBR ranging from − 1.60 log_2_FC (FDR = 9.04E-04) to − 5.59 log_2_FC (FDR = 7.42E-04). This may indicate modulation of the peptidoglycan layer by BBR treatment. Further support of this was the significant increase in expression of *murJ*, encoding Lipid II flippase (1.69 log_2_FC; *P* = 0.02; FDR =0.16) and *murR* transcriptional regulator (1.53 log_2_FC; *P* = 1.90E-03; FDR = 0.04) with a trend for *murG* (1.12 log_2_FC; *P* = 0.10; FDR = 0.39) and *murF* (0.90 log_2_FC; *P* = 0.01; FDR = 0.12) was observed. Thus, *C. hiranonis* gene expression reflects responsiveness to bile acid-induced stress during BBR treatment.

### Clostridium hylemonae

**Fig. 8:**
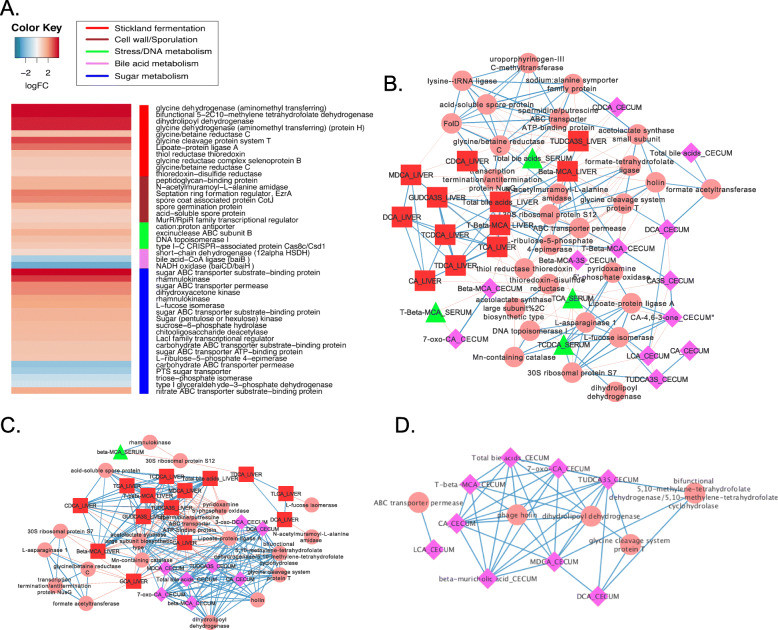
Network analysis between cecal gene expression by *Clostridium hylemonae* and bile acid metabolome. **a**. Heat map of differentially expressed genes (log_2_FC > (−)0.58; *P* < 0.05) by *C. hylemonae* in the mouse cecum between control diet and berberine treatment. **b**. Network displaying interactions between expressed genes and bile acid metabolome in control mice. **c**. Network displaying interactions between expressed genes and bile acid metabolome in berberine-treated mice. **d**. Sub-network of cecal bile acid-gene expression network from berberine-treated mice. Data points with Spearman’s correlations < 0.7 and a *P* values < 0.05 are displayed

BBR differentially regulated 92 genes in *C. hylemonae.* Of note, a gene predicted to encode the septation ring formation regulator, EzrA, was among the most highly up-regulated genes (2.41 log2FC; *P* = 1.39E-03) (Fig. 8a; Supplementary Dataset), but did not correlate with cecal bile acids. Specifically, genes involved in bile acid 7α-dehydroxylation by *C. hylemonae* were down-regulated, including *baiB* encoding bile acid coenzyme A ligase (1.45 Log_2_FC; *P* = 0.04), and *baiCD* encoding bile acid NAD-dependent 3-dehydro-4-oxidoreductase (− 2.42 Log_2_FC; *P* = 3.61E-03) (Fig. 8a). Phage genes, including holin (2.10 log_2_FC; *P* = 2.8E-04) and siphovirus DUF859 (1.96 Log_2_FC; *P* = 1.27E-3), as well as type I-C CRISPR Cas8c/Csd1 (1.09 log_2_FC; *P* = 0.04) were up-regulated by BBR. In control mice, phage holin expression was positively correlated with cecal DCA (r = 0.81; *P* = 0.021), but negatively correlated with cecal T-β-MCA (r = − 0.83; *P =* 0.016) and β-MCA in the liver (r = − 0.94; *P* = 0.0) (Fig. 8b; Supplementary Dataset). In BBR treated mice, phage holin was positively associated with total cecal bile acids (r = 1.0; *P =* 0.0) and had a strengthened positive correlation with cecal DCA (r = 0.9; *P* = 0.006) (Fig. 8c & d; Supplementary Dataset).

Cecal RNA-Seq analysis revealed two polycistronic operons involved in the Stickland fermentation of glycine, including the glycine dehydrogenase and glycine reductase pathway genes and the formation of cofactors such as lipoate that were significantly up-regulated by BBR (Fig. 8a). In control ceca, expressed genes involved in glycine reductase (*FolD*, *grdD*) appeared to be indirectly and negatively correlated to bile acids via transcription terminator/antiterminator NusG (Fig. 8b). Lipoate-protein ligase A expression was positively correlated with expression of metabolic genes as well as CA-4,6–3-one. In BBR treated mice, lipoate-protein A displayed strong positive correlation with total (r = 0.8; *P* = 0.046) and individual cecal bile acids, such as DCA (r = 0.9; *P* = 0.006), as well as weak negative correlations with liver bile acids (Fig. 8c). Positive correlations were observed between lipoate-protein ligase A and FolD, glycine cleavage protein T, and dihydrolipoyl dehydrogenase, indicating that the significant increase in glycine metabolism with BBR treatment was at least partially driven by increased bile acid concentration in the cecum.

Genes involved in sporulation in *C. hylemonae* including spore coat associated protein (*cotJA*; 2.29 log2FC; *P =* 2.89E-04), *N*-acetylmuramoyl-L-alanine amidase (*cwlD*; 1.54 log2FC; *P* = 0.03), spore germination protein (1.43 log2FC; *P* = 0.03), acid-soluble spore protein (0.96 log2FC; *P* = 0.04) were observed in response to BBR treatment. Acid-soluble spore protein was negatively correlated in control mice with liver bile acids, whereas BBR treatment resulted in a positive correlation with liver bile acids. This may indicate that up-regulation of genes involved in cell-wall maintenance and metabolism reflects the effects of both BBR and bile acids.

## Discussion

BBR treatment leads to increased conversion of cholesterol into bile acids, resulting in decreased blood cholesterol levels, since bile acid synthesis is the major route of cholesterol excretion in the body [21]. These lipid lowering effects have been confirmed in a previous meta-analysis of 27 clinical trials, thus making BBR an attractive alternative for dyslipidemic patients unable to take statins [22]. However, as a nutraceutical, BBR is not regulated with the same rigor as pharmaceutical interventions. In addition, BBRs mechanism of action (increased bile acid secretion to the GI tract), is commonly associated with negative physiological effects, including increased risk of colorectal cancer [23]. This is paradoxical given that along with demonstrated lipid lowering effects, BBR appears to exert cytotoxic effects in cancer cells [24]. Therefore, understanding BBR versus bile acid dependent effects on the gut microbiome is necessary in order to develop targeted pharmacological treatments that mimic BBRs lipid lowering outcomes. Numerous studies demonstrate that BBR alters the microbiome [4, 8, 9, 25]; however, responses of diverse commensal gut bacteria to BBR are largely unknown. The current study provides novel insight into the effects of dietary BBR on gut bacterial transcriptome profiles. This study is also the first to report effects of a changing bile acid metabolite profile on bacterial gene expression during BBR treatment. Our results are consistent with previous reports that BBR increases bile acid concentrations in the large intestine, but not the liver [6, 9, 21]. Increased bile acid concentrations in the GI tract have been reported to significantly affect the gut microbiome [26, 27]. Thus, the novel use of correlation networks to observe structural changes in transcriptome and metabolome interactions in response to BBR treatment allowed us to elucidate whether changes in gene expression were in response to increased cecal bile acid concentrations or the direct effects of BBR.

BBR feeding alters the structural composition of complex gut microbial communities potentially in response to increased colonic bile acid concentrations [6, 8, 9]. Treatment with BBR did not significantly alter the relative abundance of bacteria in the cecum of B4PC2 gnotobiotic mice. This was expected as members of the B4PC2 community are “bile-tolerant”, thus less likely to become perturbed in bile rich conditions, and growing with limited pressure for niche competition due to consortium simplicity and gnotobiotic conditions. Consequently, this provides an excellent framework by which to examine transcriptional changes of each bacteria in response to BBR treatment. Use of correlation networks to analyze transcriptomic and metabolomic changes in this minimal community allows us to determine functional changes in these bacteria that are responses to bile acid concentrations versus direct effects of BBR on member composition or abundance.

Indeed, BBR did significantly alter gut microbial-bile acid metabolite interactions (Fig. 2**;** Supplementary Dataset Correlation Networks). For example, sulfated bile acids were positively correlated to bile acid 7α-dehydroxylating bacteria, *C. hiranonis* and *C. hylemonae*, and negatively correlated with Bacteroidetes spp. The host sulfates bile acids to act as signaling molecules, and sulfation acts as the major pathway by which humans detoxify hydrophobic bile acids [28]. However, currently little is known about the effects of sulfated-bile acids on anaerobic bacterial physiology. Our results indicate a potential relationship between sulfated bile acids and microbial physiological changes.

While our study was not designed to address the metabolism of BBR in gnotobiotic mice, it was previously shown that microbial reduction of BBR to dihydroberberine by flavin mononucleotide (FMN)-dependent nitroreductase was necessary to facilitate host BBR absorption [5]. Indeed, BBR up-regulated an FMN-dependent nitroreductase in *B. uniformis***,** which may indicate metabolism of BBR by the B4PC2 community. Numerous BBR metabolites have been reported in animal models [29], and it is probable that additional anaerobic bacteria and microbial enzymes will be identified that generate BBR derivatives.

We observed a negative correlation between *B. wadsworthia* and the cecal secondary bile acids DCA and 3-oxo-DCA in control and BBR treatment, respectively. Correlations of bile acid metabolites and differential transcripts expressed by *B. wadsworthia* indicate DCA induces DNA repair and universal stress protein which controls expression of a number of genes involved in redox reactions and electron transport. In particular, a glycyl radical enzyme encoding gene, recently reported to be involved in taurine respiration [18], was positively associated with DCA in the cecum. Interestingly, an ethanolamine degradation pathway was highly up-regulated in *B. wadsworthia* along with the nitrate reductase γ-subunit (Fig. 3d). A previous metabolomic study of BBR and oryzanol demonstrated a significant increase in fecal ethanolamine with 4-week treatment of 150 mg kg^− 1^ BBR [30]. Phosphotidylethanolamine is the primary membrane lipid in bacteria [31] and gut microbes have evolved complex pathways to metabolize this compound. The antimicrobial nature of BBR leads to lysis and release of bacterial membrane components as evidenced by prior descriptions of reduced total microbial load by BBR [8], and reports that BBR inhibits the cell division protein FtsZ thus leading to cell death [32, 33]. Metatranscriptomic analysis indicates that *B. wadsworthia* up-regulates a lipid transporter (LadL) and genes predicted to encode enzymes involved in ethanolamine utilization. Metabolism of bacterial phosphatidylethanolamine by gut bacteria would yield ATP by substrate-level phosphorylation from acetyl-phosphate [26]. Additionally, genes encoding enzymes in the citric acid cycle and the electron transport chain were up-regulated, which may indicate that *B. wadsworthia* converts bacterial fatty acids to acetyl-CoA via anaerobic respiration, with nitrate and taurine serving as terminal electron acceptors. Nitrate reductase induction is intriguing since other pathobionts, such as enterohemorrhagic *Escherichia coli,* utilize host nitrosative respiratory bursts for anaerobic respiration [34]. These results indicate that increased concentration of secondary bile acids due to BBR treatment induces the stress response in *Bilophila,* and that BBR may directly affect microbial physiology through alteration of growth substrates and terminal electron acceptors used in anaerobic respiration.

There were several important observations made with respect to the effect of BBR on *C. hylemonae*. First, numerous cell wall and membrane architecture genes were differentially regulated (Fig. 7d). In particular, the regulator of septation ring formation, EzrA, was significantly up-regulated by BBR (2.41 log2FC; *P* = 1.39E-03). Previous work in *E. coli* demonstrated that BBR inhibits GTPase activity and destabilizes septation ring protofilaments [32]. This indicates that BBR may affect microbial growth through targeting EzrA in both gram-negative and gram-positive bacteria inhabiting the GI tract [33]. Importantly, EzrA transcripts were not observed to correlate with bile acid metabolites, suggesting that inhibition of EzrA gene expression may be directly due to BBR treatment as seen in *E. coli.* By contrast, BBR treatment led to a tight clustering of cecal bile acids to phage holin in *C. hylemonae* (Fig. 7c), suggesting that BBR-induced alterations in the gut microbiome observed in complex consortia may be partly due to induction of the phage lytic cycle through bile acid toxicity. Indeed, previous studies have shown that bile acids induce phage lytic cycle in intestinal pathogens [35, 36].

Na+/H+ antiporter was up-regulated by BBR and highly correlated with cecal bile acids in *Ba. vulgatus* (Fig. 5), *P. distasonis* (Fig. 6), *C. hiranonis* (Fig. 7), and *C. hylemonae* (Fig. 8). This is consistent with previous reports of bile resistance by the multi-subunit Na+/H+ antiporter in *Bacillus subtilis* [37] and *Vibrio cholera* [38]. Thus, the process by which BBR alters the gut microbiome is likely partly due to its choleretic effects. Previous studies in which bile acids were fed to rodents, and thus enriched in the GI tract, demonstrate the role bile acids play in structuring the gut microbiome via anti-microbial selection pressure [26].

## Conclusions

The current study indicates that BBR has both bile acid-dependent and independent effects on the B4PC2 consortium related to stress response, bile and xenobiotic tolerance, and changes in energy metabolism. These responses observed in a defined human gut consortium in gnotobiotic mice are critical to elucidate the effects of BBR supplementation on complex gut microbial communities. The implications of this research are increased understanding of altered microbial function in response to BBR versus increased GI concentrations of bile acid, which may lead to targeted pharmaceutical interventions that mimic the positive effects observed with supplementation of the nutraceutical BBR.

## Methods

### Bacterial strains and chemical reagents

The B4PC2 consortium consisted of *Bacteroides uniformis* ATCC 8492, *Bacteroides vulgatus* ATCC 8482, *Clostridium hylemonae* DSM 15053, *Clostridium hiranonis* DSM 13275, *Parabacteroides distasonis* DSM 20701, *Bilophila wadsworthia* DSM 11045, and *Blautia producta* ATCC 27340. Strains were cultured and stored as previously described [12]. Authentic reference bile acids were described in our recent publication [12] and purchased from Sigma-Aldrich (St. Louis, MO) and internal standards were obtained from C/D/N Isotopes (Pointe-Claire, QC, Canada). Rare bile acids and sulfated-derivatives were gifts from Professor Iida, Nihon University, Tokyo, Japan (takaiiada@chs.nihon-u.ac.jp). Solvents (water, ethanol, methanol, acetonitrile) were of high-performance liquid chromatography grade, and ammonium acetate was analytical grade, all of which were purchased from Kanto Chemical (Tokyo, Japan).

### Gnotobiotic mice

All experiments were approved by the Institutional Animal Care and Use Committees of the Mayo Clinic (Rochester, MN) (Protocol# A00001902–16). Mice were provided ad libitum access to autoclaved LabDiet 5 K67 through wire bar feeders. Ad libitum access to autoclaved water was provided by means of polysulfone bottles with a shoulder hole. Six-week old C57BL/6 N mice (*N* = 12; Taconic Farms, Germantown, NY) were randomly separated into two isolators (3 males/3 females per isolator) and inoculated with the B4PC2 consortium as previously described [13]. From day [14, 39–51], mice were gavaged daily with either sterile saline, or BBR (100 mg kg^− 1^ final). Berberine for oral gavage (25 mg/ml) was suspended in PBS containing 0.5% carboxymethylcellulose to maintain solubility. Mice were euthanized on day 27 by CO_2_ asphyxiation followed by cervical dislocation, and content for bile acid and microbial community analysis were collected and stored as previously described [13].

### Microbiome community profiling

Genomic DNA was extracted from cecum samples and library preparation, pooling, and MiniSeq sequencing were performed at the DNA Services facility, Research Resources Center, University of Illinois at Chicago as described previously [13]. Genomic DNA was PCR amplified with primers 515F-modified and 926R that contained 5′ common sequence tags [13] using a two-stage “targeted amplicon sequencing” protocol [40–42]. First and second stage PCR amplifications were performed in 10 μl reactions in 96-well plates, using the MyTaq HS 2X mastermix (Bioline, Taunton, MA), and PCR conditions as recently described [13]. Pooled libraries were purified with an AMPure XP cleanup protocol, spiked with phiX, and subjected to MiniSeq sequencing to obtain 2 × 150 bp paired-end reads. Forward and reverse reads were merged using PEAR [43] and trimmed based on a quality threshold of *p* = 0.01. Ambiguous nucleotides and primer sequences were removed and sequences less than 300 bp were discarded. Chimeric sequences were identified and removed using the USEARCH algorithm with a comparison to GreenGenes 13_8 [14, 44]. Resulting sequence files were merged with sample information and operational taxonomic unit clusters were generated in QIIME using the UCLUST algorithm with a 97% similarity threshold [14, 45]. Taxonomic annotations for each OTU were determined using the UCLUST algorithm and GreenGenes 13_8 reference with a minimum similarity threshold of 90% [14, 44].

#### Cecal RNA-Seq analysis

Extraction, library preparation and sequencing were performed at the DNA Services facility, Research Resources Center, University of Illinois at Chicago, as previously described [13]. Cecal tissue was homogenized and total RNA was extracted from mouse cecum using an EZ1 RNA tissue kit (Qiagen, Germantown, MD) [13]. Two hundred and fifty ng of total RNA was double depleted and utilized to generate cecal mRNA-Seq libraries using a ScriptSeq v2 RNA-Seq Library Prep kit (Illumina). Pooled libraries were then sequenced on an Illumina NextSeq500 instrument using paired-end 2 × 150 base reads. Bioinformatics of RNA-Seq datasets was performed as previously described [13]. Raw RNA-seq reads with Q scores < 32 were aligned with Ribosomal RNA sequences prepared from the B4PC2 genomes using bowtie2 (v2.3.3.1). HTSeq (v0.9.1) counting was performed in union mode against Gene Feature Format annotations of the B4PC2 genomes and compared to coding DNA sequences of each bacterium. Differential gene expression analysis between BBR treatment and control was performed using edgeR [46] and limma [47] R packages, with a minimum *P*-value of < 0.05 accepted as indicating differentially expressed genes. Genes were binned according to known functionality, and category analysis was performed using eggNOG [48].

#### Sample preparation for bile acid metabolomics

Bile acid sample preparation and LC-MS/MS for bile acid analysis were essentially based on the previously developed method and was performed after extraction from samples as previously described [13, 49]. In short, cecum contents were lyophilized and 90% ethanol (2 ml) was added to 10 mg of the dried matter. For liver, 300–400 mg of sample was homogenized with cold water (500 μl) and 20 mg/ml of Proteinase K solution, and digested at 55 °C for 16 h. Bile acids were extracted from dried cecal content and homogenized liver three times by ultra-sonication at room temperature for 1 h. Supernatant was separated by centrifugation at 2500 rpm for 5 min after each ultra-sonication cycle and combined into a glass test tube. Liver and cecal samples were then evaporated to dryness under an N_2_ stream. Serum (50 μl) was added to acetonitrile (5 ml) and was also evaporated to dryness. Prepared crude bile acid extracts were then re-suspended in 90% ethanol (1 ml) by ultra-sonication and, deuterium-labeled internal standards, *d*_4_-CA, *d*_4_-GCA and *d*_4_-TCA were added at 100 nmol/ml. A diluted aliquot was applied to a GL Sciences InertSep C18-B solid-phase extraction cartridge (100 mg/ml; Tokyo, Japan), washed with water, eluted with 90% ethanol, and dried to remove solvent. The remaining residue was dissolved in 20% acetonitrile, and an aliquot of the solution was analyzed by LC/ESI-MS/MS.

#### LC/ESI-MS/MS analysis

LC/ESI-MS/MS analysis was conducted as recently described using an LCMS-8050 tandem mass spectrometer, equipped with an ESI probe and Nexera X2 ultra high-pressure liquid chromatography system (Shimadzu, Japan). Linear gradient elution on a InertSustain C18 (150 mm × 2.1 mm ID, 3 μm particle size; GL Sciences Inc., Tokyo, Japan) separation column was employed at a flow rate of 0.2 ml/min at 40 °C. Mobile phase, LC parameters and MS parameters were the same as recently reported [13].

#### Network correlation analysis

Correlation network analysis [50] and Correlation Difference Network analysis were performed for cecal transcriptomics and bile acid metabolomics from serum, liver, and cecum. Data were combined into a single feature table and Spearman correlations were calculated between all features using a custom Python program and *P* values were calculated as described previously [51]. A PERL script was used to filter the correlations based on a defined Rho (i.e. r > 0.7) and a defined *P*-values (i.e. *P* > 0.001). Networks were plotted in Cytoscape to visualize the statistically significant correlations and these are used to develop hypotheses about the interactions between the features [52]. We then used a custom Python program to calculate correlation differences [53] between the feature pairs; that is correlations that have significantly (*P <* 0.01) changed between the two treatments. This allows inferences of interactions that have changed between the control and BBR treatment identifying key metabolic shifts induced by BBR. The Correlation Network and Correlation Difference tools are deployed on our Galaxy Portal (http://mbac.gmu.edu:8080).

#### Accession numbers

Cecal RNA-Seq datasets were deposited as Bioproject PRJNA523415.

## Supplementary Information

**Additional file 1 Fig. S1.** Profile of liver bile acids from control and berberine treated mice.

**Additional file 2 Fig. S2.** Profile of three most abundant liver bile acids from control and berberine treated mice.

**Additional file 3 Fig. S3.** Serum bile acid profile in control and berberine treated mice.

**Additional file 4 Fig. S4.** Profile of cecal bile acids between control and berberine treated mice not represented in Fig. 1. Significance determined by student *t* test. * *P* < 0.05.

**Additional file 5 Fig. S5.** 16S rDNA profile of human gut bacterial consortium in cecal samples of gnotobiotic fed control diet versus berberine. A. Relative abundance of bacterial families in control mice (C1-C6) and berberine treatment (B1-B6) B. Non-metric multidimensional scaling (NMDS) plot of beta diversity based on Bray-Curtis index. ANOSIM test results: *R* = 0.141, *P* = 0.123, 999 permutations.

**Additional file 6 Fig. S6.** Shannon Index comparison between control mice and berberine treatment. The rarified 23,900 MiSeq dataset was used. Mann-Whitney test *P* = 0.309.

**Additional file 7.** Supplementary Dataset 1.

**Additional file 8.** Supplementary Dataset 2.

**Additional file 9.** Supplementary Dataset 3.

**Additional file 10.** Supplementary Dataset 4.

## Data Availability

All data generated or analyzed during this study are included in this published article [and its supplementary information files]. Cecal RNA-Seq datasets were deposited as Bioproject PRJNA523415.

## Abbreviations

BBR: Berberine
B4PC2 consortium: *Bacteroides vulgatus, Bacteroides uniformis. Bilophila wadsworthia, Blautia producta, Parabacteroides distasonis, Clostridium hylemonae, Clostridium hiranonis*
PBS: Phosphate buffered saline
CFU: Colony forming units
TAS: Targeted amplicon sequencing
NMDS: Non-metric multidimensional scaling
ANOSIM: Analysis of similarities
TCA: Taurocholic acid
CA: Cholic acid
GCA3S: 3-sulfoglycocholic acid
TCA3S: 3-sulfotaurocholic acid
CA3S: 3-sulfo cholic acid
GCDCA: Glycochenodeoxycholic acid
TCDCA: Taurochenodeoxycholic acid
CDCA: Chenodeoxycholic acid
GCDCA3S: 3-sulfoglycochenodeoxycholic acid
TCDCA3S: 3-sulfo-taurochenodeoxycholic acid
CDCA3S: 3-sulfochenodeoxycholic acid
GUDCA: Glycoursodeoxycholic acid
TUDCA: Tauroursodeoxycholic acid
UDCA: Ursodeoxycholic acid
GUDCA3S: 3-sulfoglycoursodeoxycholic acid
TUDCA3S: 3-sulfotauroursodeoxycholic acid
UDCA3S: 3-sulfoursodeoxycholic acid
GDCA: Glycodeoxycholic acid
TDCA: Taurodeoxycholic acid
DCA: Deoxycholic acid
GDCA3S: 3-sulfoglycodeoxycholic acid
TDCA3S: 3-sulfotaurodeoxycholic acid
DCA3S: 3-sulfodeoxycholic acid
GI: Gastrointestinal
GLCA: Glycolithocholic acid
TLCA: Taurolithocholic acid
LCA: Lithocholic acid
GLCA3S: 3-sulfoglycolithocholic acid
TLC3S: 3-sulfotaurolithocholic acid
LCA3S: 3-sulfolithocholic acid
GHCA: Glycohyocholic acid
THCA: Taurohyocholic acid
HCA: Hyocholic acid
nor-CA: Norcholic acid
MCA: Murocholic acid
T-α-MCA: Tauro-α-murocholic acid
α-MCA: α-murocholic acid
T-α-MCA-3S: Tauro-α-murocholic acid 3-sulfate
α-MCA-3S: α-murocholic acid 3-sulfate
T-β-MCA: Tauro-β-murocholic acid
β-MCA: β-murocholic acid
T-β-MCA-3S: Tauro-β-murocholic acid 3-sulfate
β-MCA-3S: β-murocholic acid 3-sulfate
T-β-MCA: Tauro-ω-murocholic acid
MDCA: Murodeoxycholic acid
GHDCA: Glycohyodeoxycholic acid
THDCA: Taurohyodeoxycholic acid
HDCA: Hyodeoxycholic acid
iso-CA: Iso-cholic acid
3-oxo-CA: 3-oxo-cholic acid
7-oxo-DCA: 7-oxo-deoxycholic acid
12-oxo-LCA: 12-oxo-chenodeoxycholic acid
iso-CDCA: Isochenodeoxycholic acid
3-oxo-CDCA: 3-oxo-chenodeoxycholic acid
7-oxo-LCA: 7-oxolithocholic acid
UCA: Ursocholic acid
iso-DCA: Iso-deoxycholic acid
3-oxo-DCA: 3-oxo-deoxycholic acid
iso-LCA: Iso-lithocholic acid
allo-iso-LCA: Allo-iso-lithocholic acid
