# Supplementary figures and images for: Berberine alters gut microbial function through modulation of bile acids
Source: BMC Microbiol. 2021 Jan 11;21:24. doi: 10.1186/s12866-020-02020-1 (PMC7798349; doi:10.1186/s12866-020-02020-1)

# Liver

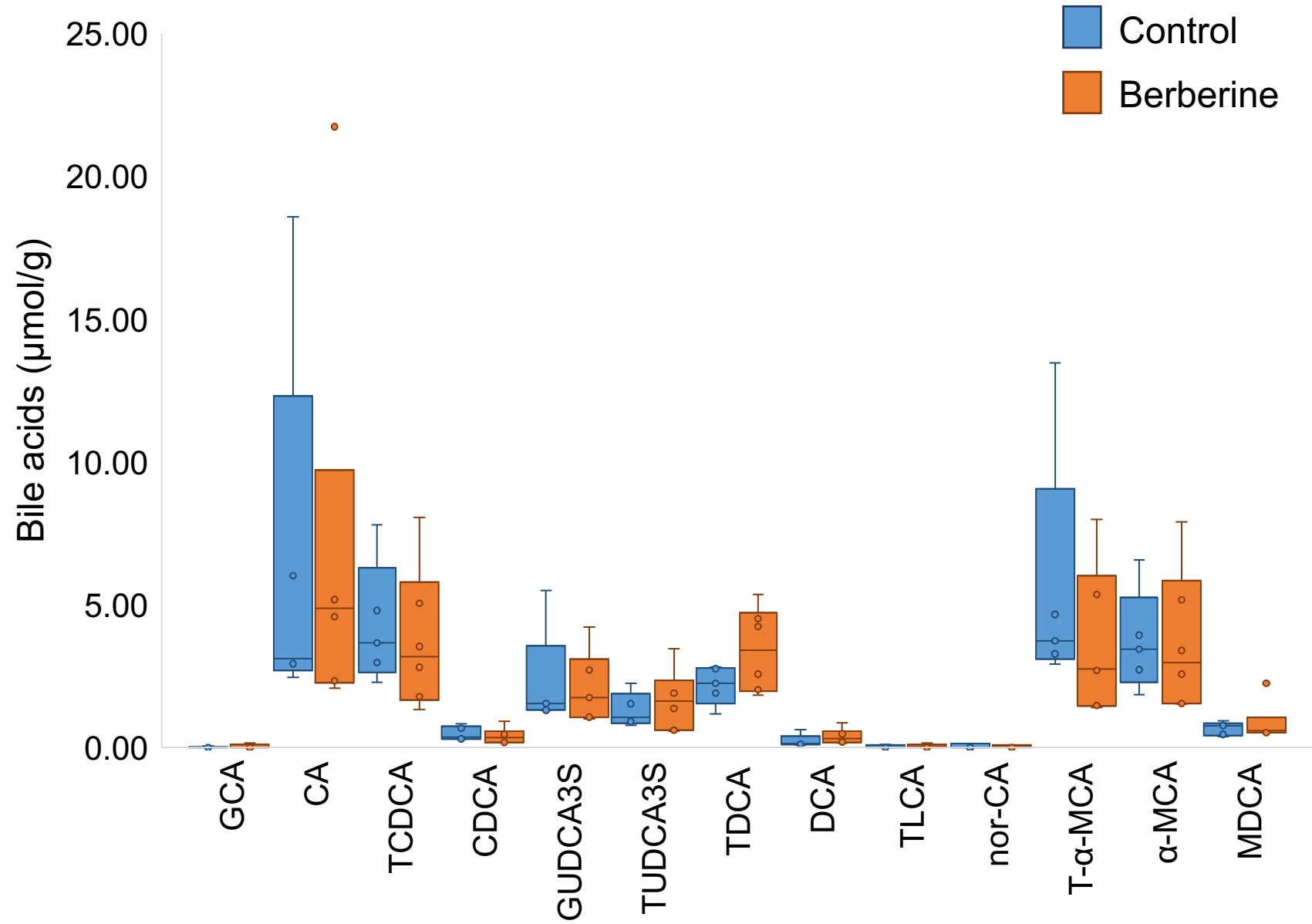

Supplement: Supplementary file 1 — Additional file 1 Fig. S1. Profile of liver bile acids from control and berberine treated mice. [file 12866_2020_2020_MOESM1_ESM.pdf]

# Liver

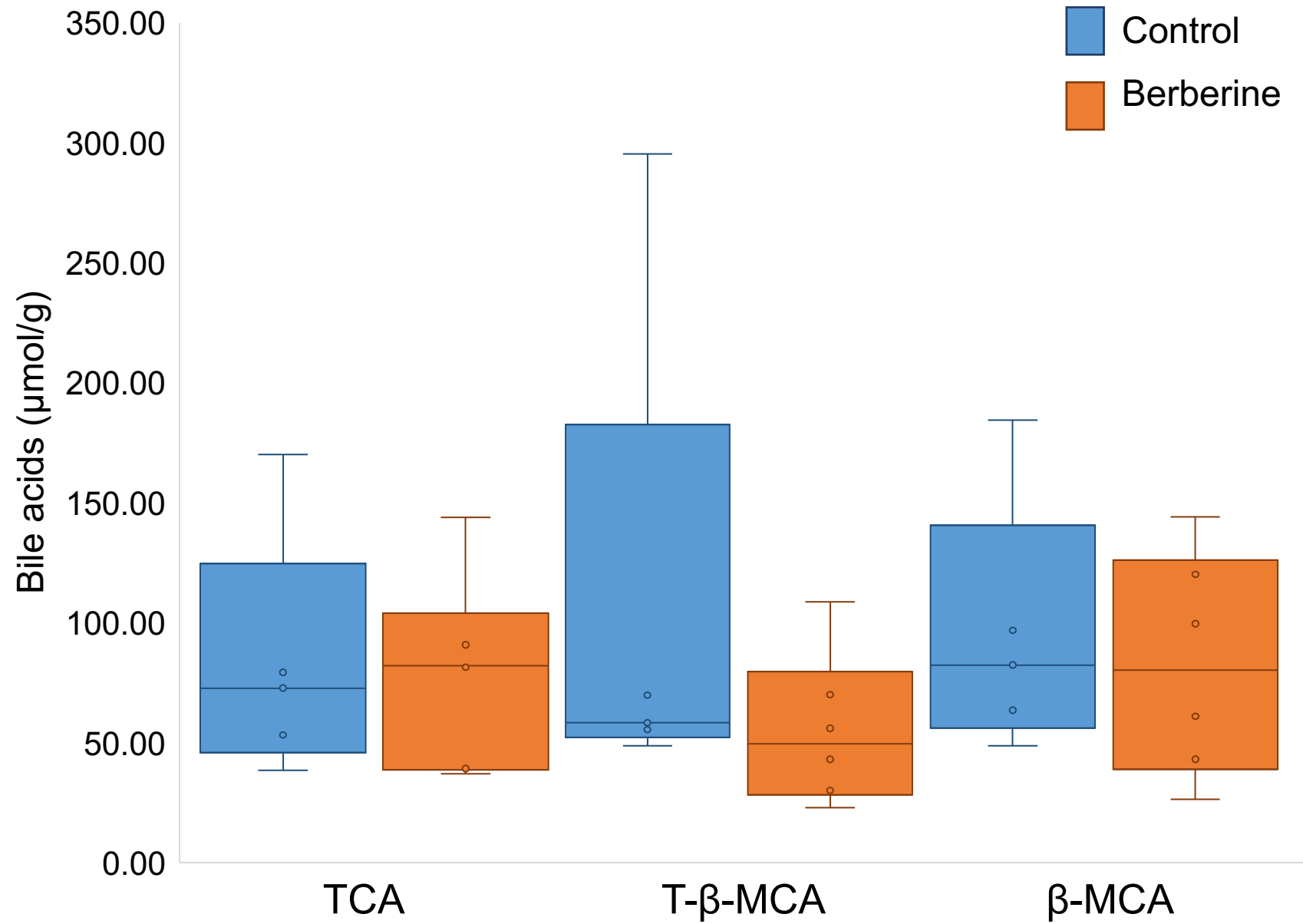

Supplement: Supplementary file 2 — Additional file 2 Fig. S2. Profile of three most abundant liver bile acids from control and berberine treated mice. [file 12866_2020_2020_MOESM2_ESM.pdf]

Serum

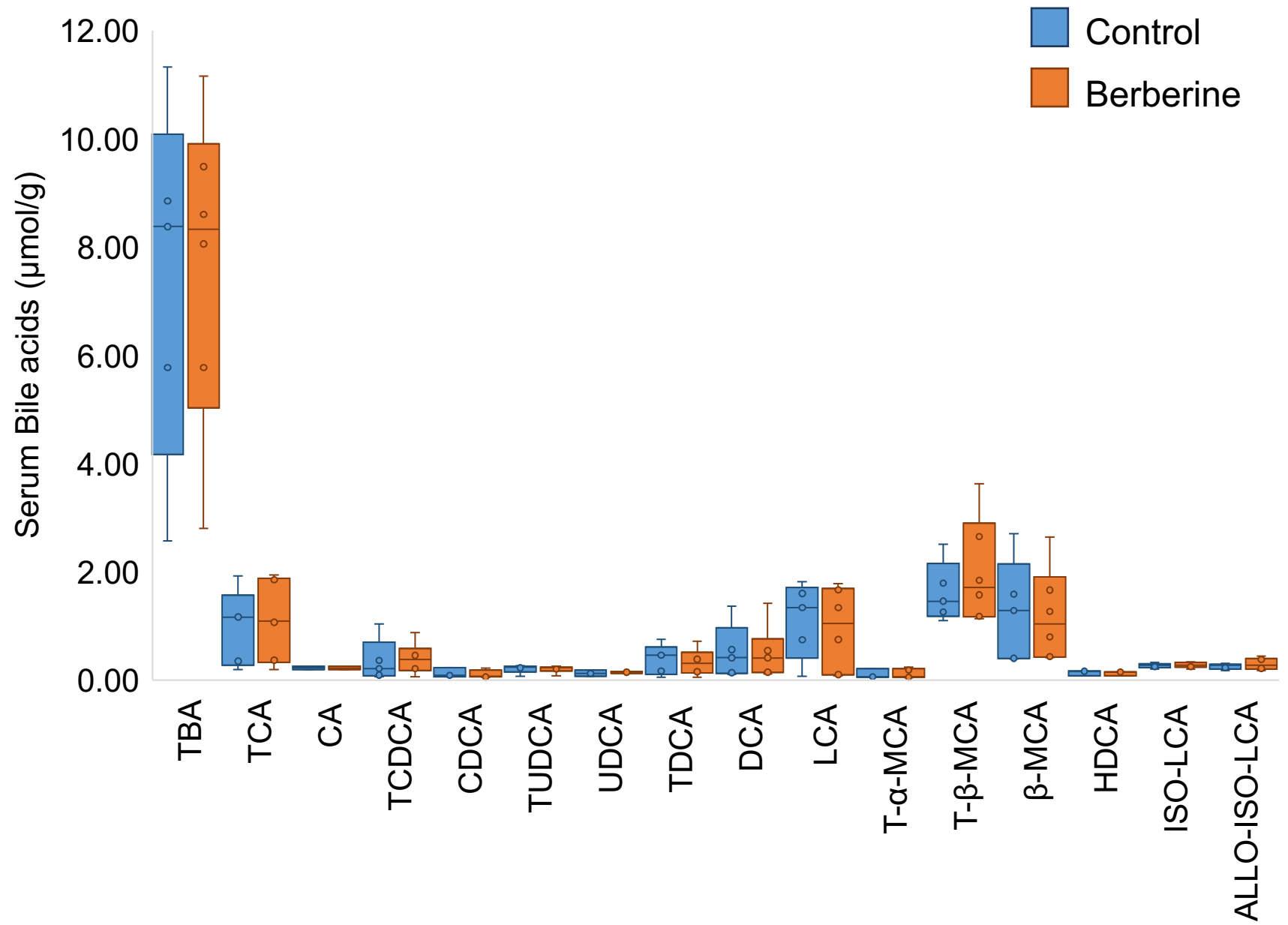

Supplement: Supplementary file 3 — Additional file 3 Fig. S3. Serum bile acid profile in control and berberine treated mice. [file 12866_2020_2020_MOESM3_ESM.pdf]

# Cecum

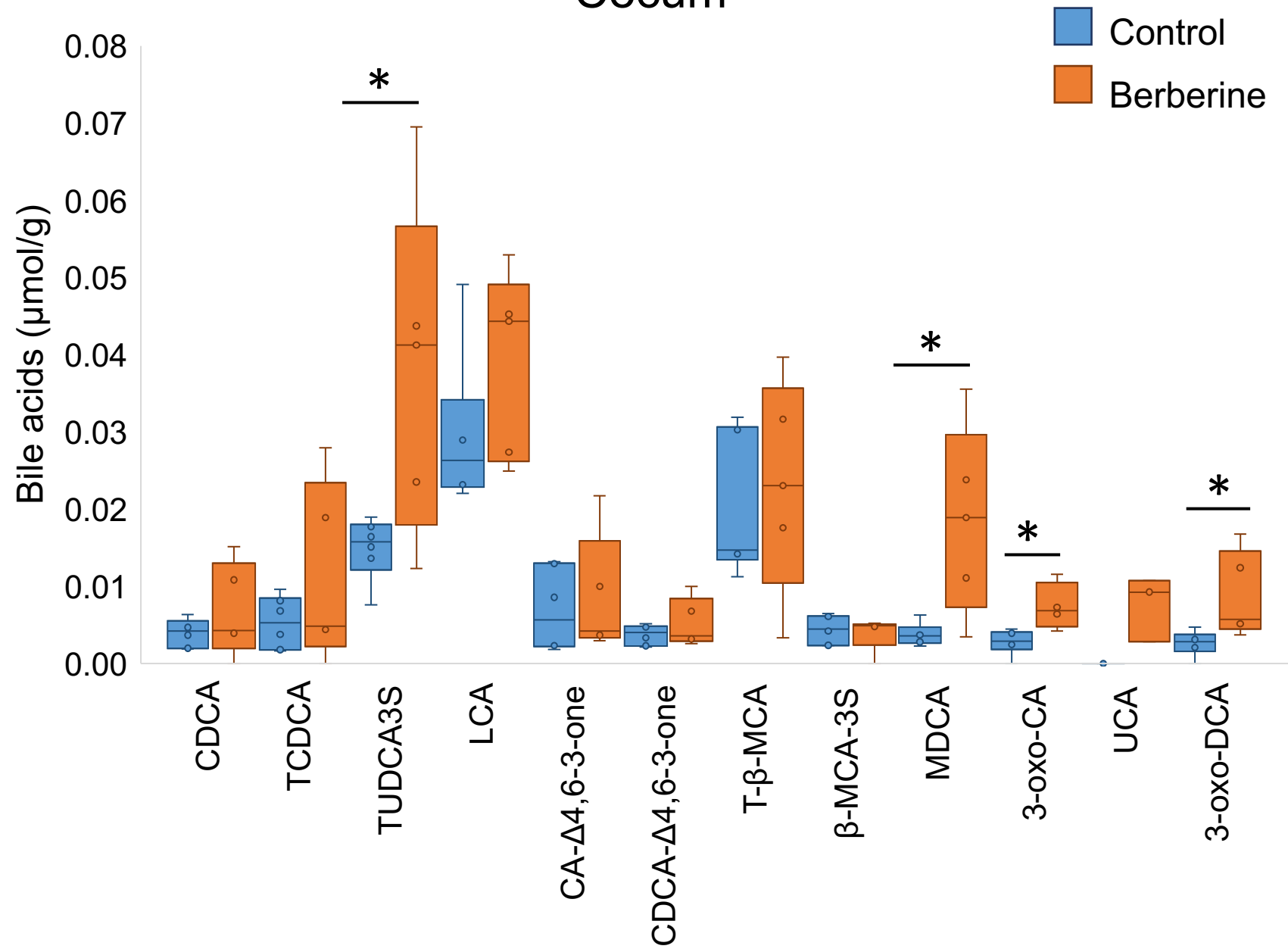

Supplement: Supplementary file 4 — Additional file 4 Fig. S4. Profile of cecal bile acids between control and berberine treated mice not represented in Fig. 1. Significance determined by student t test. * P < 0.05. [file 12866_2020_2020_MOESM4_ESM.pdf]

A.

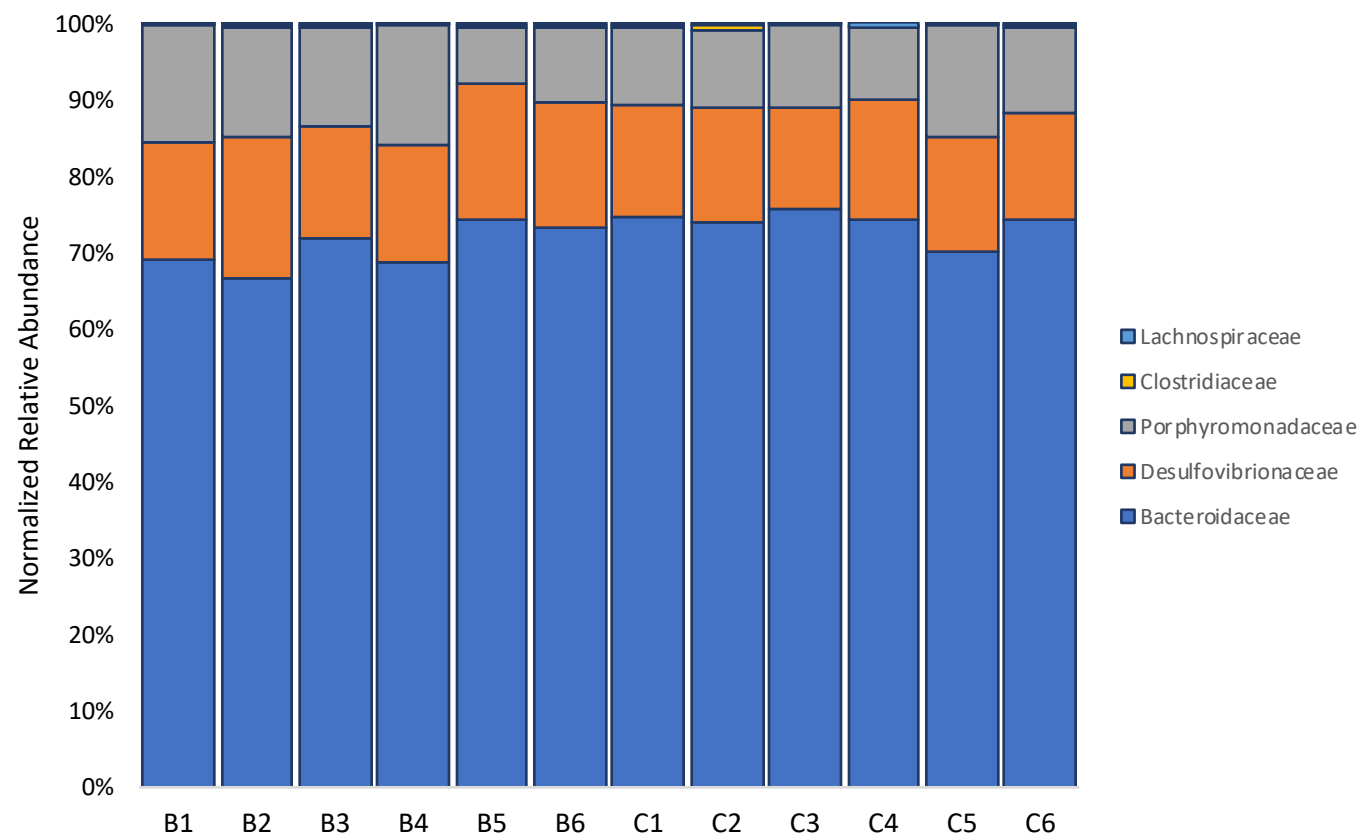

B.

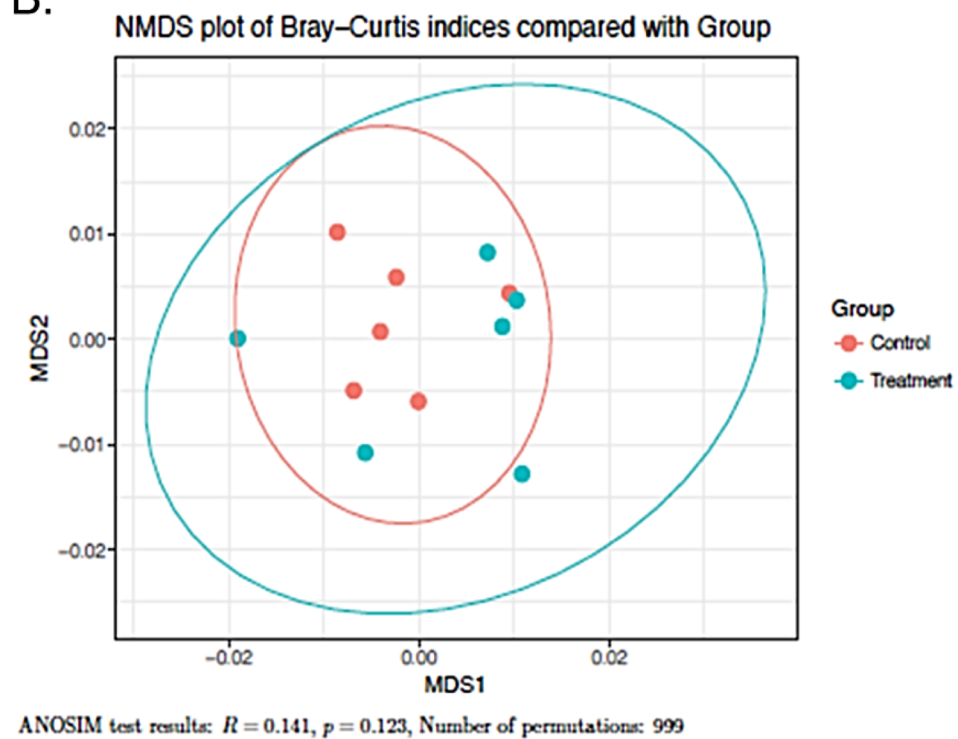

Supplement: Supplementary file 5 — Additional file 5 Fig. S5. 16S rDNA profile of human gut bacterial consortium in cecal samples of gnotobiotic fed control diet versus berberine. A. Relative abundance of bacterial families in control mice (C1-C6) and berberine treatment (B1-B6) B. Non-metric multidimensional scaling (NMDS) plot of beta diversity based on Bray-Curtis index. ANOSIM test results: R = 0.141, P = 0.123, 999 permutations. [file 12866_2020_2020_MOESM5_ESM.pdf]

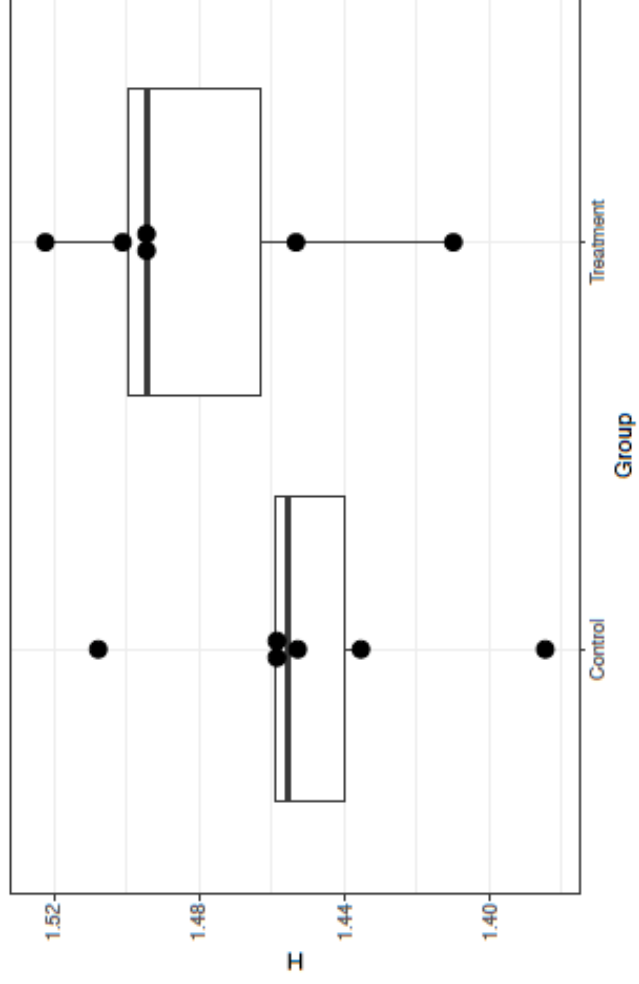

Supplement: Supplementary file 6 — Additional file 6 Fig. S6. Shannon Index comparison between control mice and berberine treatment. The rarified 23,900 MiSeq dataset was used. Mann-Whitney test P = 0.309. [file 12866_2020_2020_MOESM6_ESM.pdf]
